# The Dynamic Remodeling of Plant Cell Wall in Response to Heat Stress

**DOI:** 10.3390/genes16060628

**Published:** 2025-05-24

**Authors:** Chengchen Lu, Wenfei Li, Xiaomeng Feng, Jiarui Chen, Shijie Hu, Yirui Tan, Leiming Wu

**Affiliations:** The National Engineering Laboratory of Crop Resistance Breeding, School of Life Sciences, Anhui Agricultural University, Hefei 230036, China; luchengchen1210@163.com (C.L.);

**Keywords:** cell wall, heat stress, cell wall integrity, cellulose, pectin

## Abstract

Heat stress has a significant negative impact on plant growth, development, and yield. The cell wall, a key structural feature that sets plants apart from animals, not only acts as the first physical barrier against heat stress but also plays an active role in the heat stress (HS) response through signaling pathways. The plant cell wall has a complex structural composition, including cellulose, hemicellulose, lignin, and pectin. These components not only provide mechanical support for cell growth but also constitute the material basis for plant response to environmental changes. This review summarizes recent research on how the cell wall’s structural composition affects its mechanical properties in response to stresses. It examines changes in plant cell walls under HS and the adaptive mechanisms leading to cell wall thickening. Additionally, it explores the role of cell wall integrity in sensing and transmitting HS, along with the molecular mechanisms that maintain this integrity. Finally, it addresses unresolved scientific questions regarding plant cell wall responses to HS. This review aims to provide a theoretical foundation and research direction for enhancing plant thermotolerance through genetic improvement of the cell wall.

## 1. Introduction

Temperature variation is a key environmental factor influencing plant growth, development, distribution, and yield [1]. Elevated temperatures (e.g., 24–30 °C) can accelerate the growth of many crops, such as maize and rice. However, excessive heat (e.g., above 32 °C) can cause cumulative damage to plant growth and development [2]. Short-term heat stress (HS) can disrupt physiological processes like photosynthesis and respiration, resulting in symptoms such as leaf yellowing and wilting. Under medium-term HS, plants may show significant growth inhibition, delayed flowering, and impaired fruit development. If high temperature (HT) persist for weeks, plants may suffer from severe water shortages, nutrient imbalances, and even death. Therefore, HS may occur at different stages of plant growth, causing damage to different organs of the plant, such as leaves, roots, stomata, pollen, and fruit, ultimately leading to a decrease in plant yield and quality [3]. Yields of staple crops are more susceptible to HS than those of other non-domesticated plants. For example, for every 1 °C increase in the global average temperature, the yields of staple food crops such as wheat, rice, maize, and soybean will decrease by 6.0%, 3.2%, 7.4%, and 3.1%, respectively [4].

To minimize the growth damage caused by HS, plants have evolved a complex set of molecular mechanisms for sensing and responding to HS [5]. The cytoplasmic membrane is a critical site for transmitting and sensing HS signals, possessing several types of calcium ion (Ca^2+^) channels and receptor-like kinases [6]. HS can trigger changes in enzyme activities associated with cell wall modification. For instance, the activation of pectin methylesterases (PMEs) promotes the loosening of the cell wall, facilitating the influx of Ca^2+^ into the cytoplasm. This increase in cytoplasmic Ca^2+^ concentration acts as a signal to subsequently initiate the plant’s response to HS [7]. In addition, HS leads to a high intracellular accumulation of reactive oxygen species (ROS), which are important signaling molecules. Plants scavenge excess ROS by activating a series of genes, including the gene encoding ascorbate peroxidase APX (Ascorbate Peroxidase) [3]. To adapt to HT environment, plants activate the expression of heat shock proteins (HSPs), which have multiple functions in HS response and regulation. HSPs not only act as chaperones to renature misfolded proteins that are important for thermotolerance but also form complexes with heat shock transcription factors (HSFs) to regulate the expression of downstream genes [7,8].

In recent years, dynamic changes in plant cell walls have played an important role in sensing and responding to adversities such as high salt and HT [5,9,10]. For example, the overexpression of heat shock transcription factor 4 (*ZmHSF4*) in maize markedly upregulates the expression of the cell wall synthesis-related gene *ZmCesA2*, resulting in increased cellulose accumulation and enhanced mechanical strength of the cell wall. Consequently, this enhancement contributes to improved heat tolerance [11]. The cell wall serves both as a physical barrier to abiotic stress and participates in the transduction of stress signals through cell wall integrity receptors [10]. Although several review articles have elaborated on the molecular mechanisms of plant response to HS [2,12], there are fewer research reviews involving the dynamic changes in and remodeling mechanisms of the plant cell wall structure under HS. This paper explored how plant cell walls change structurally under HS and the molecular mechanisms behind cell wall integrity in sensing, transducing, and responding to HS. The findings provided new perspectives and a theoretical basis for an in-depth understanding of plant heat tolerance.

## 2. Structural and Mechanical Properties of Plant Cell Walls

### 2.1. Structural Properties of the Plant Cell Wall

The plant cell wall is a crucial external structure of plant cells and one of the defining features distinguishing them from animal cells. Plant cell walls have a multilayered structure and consist of three sections, including the middle lamella, primary cell wall (PCW), and secondary cell wall (SCW). The middle lamella is a pectin layer to cement the bond between two adjoining cells [13]. The primary cell wall (PCW) is a thin and flexible structure that plays a crucial role in cell elongation and expansion. In contrast, the secondary cell wall (SCW) is deposited between the PCW and the plasma membrane during cell maturation, and it primarily contributes to mechanical strength and functional specialization [14]. The two types of cell walls exhibit distinct compositional differences. The PCW is primarily composed of polysaccharides (cellulose, hemicellulose, and pectin) and minor structural proteins such as expansin. In comparison, the SCW not only contains cellulose and hemicellulose but is also enriched with lignin, a phenolic polymer [15]. The hydrophobic nature of lignin is one of the key factors that imparts waterproofing properties to the secondary cell wall, facilitating water transport, regulating transpiration, and enhancing cell wall mechanical strength and stress resistance [16]. These compositional differences grant the PCW flexibility and plasticity, whereas the SCW achieves biomechanical stability and structural rigidity.

There are differences in the structure and composition of cell walls between monocotyledonous (monocot) and dicotyledonous (dicot) plants, which may reflect their divergence in evolutionary time and adaptation to distinct environments [17]. In dicots, the vascular bundles are arranged in a ring formation, featuring distinct layers of xylem and phloem. In contrast, monocots have scattered vascular bundles, and their xylem is generally thinner and simpler [18]. Dicots typically contain higher levels of cellulose and lignin compared to monocots. While the primary component of hemicellulose in dicots is xyloglucan, monocots predominantly feature arabinoxylan [19]. The variations in the substituents on the branched chains of xylan can influence the degree of cross-linking with other components of the cell wall, ultimately affecting its mechanical strength [20].

### 2.2. Mechanical Properties of Plant Cell Wall

The plant cell wall is a nanoscale network structure composed of multiple components that form a complex dynamic network by cross-linking with each other, which determines the mechanical properties of the cell [21]. Cellulose microfibrils form a rigid skeleton through hydrogen bonding, and their alignment orientation affects the mechanical anisotropy of the cell wall [21]. Covalent cross-linking of cellulose and hemicellulose enhances network rigidity. Lignin forms an interconnected framework by cross-linking hemicellulose, thus providing structural support and stress resistance to the SCW [22]. The degree of methyl esterification of pectin affects the viscoelasticity and flexibility of the cell wall [23]. The PCW is involved in the degree of elongation and expansion of cells, while the SCW exhibits high strength and good compression resistance through a laminar arrangement of cellulose microfibrils and lignin filling [24].

**Figure 1:**
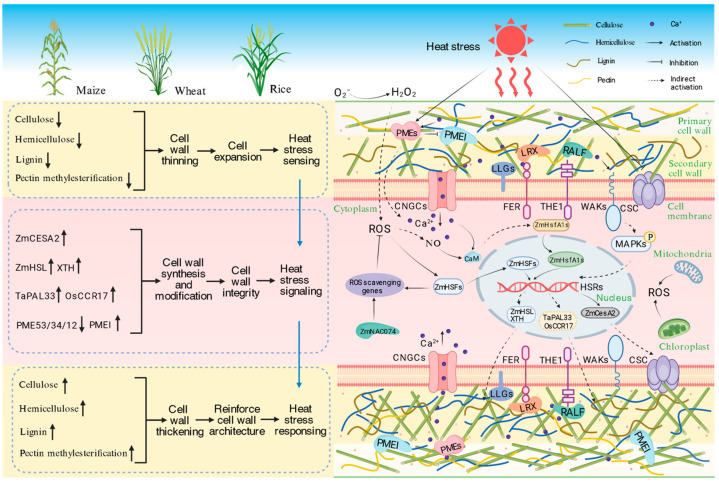
Mechanisms of plant cell wall remodeling in response to heat stress. During the initial HS perception phase, the contents of cellulose, hemicellulose, and lignin decrease, and pectin methylesterification is reduced by activating pectin methylesterase (PME) activity. These modifications serve dual physiological functions: they facilitate cell wall thinning and cellular expansion, promoting an increase in cytoplasmic Ca^2+^ concentration as an immediate adaptive response to elevated temperatures; simultaneously, they compromise cell wall integrity (CWI), leading to the generation of damage-associated signals. These signaling molecules are recognized by plasma membrane-localized receptors, triggering calcium-dependent signaling cascades that activate the expression of heat-responsive genes including transcription factors and heat shock proteins. Concurrently, HS initiates a transcriptional reprogramming that upregulates cellulose synthase genes (*ZmCESA2*), hemicellulose-modifying enzymes (ZmHSL and XTH), and lignin biosynthesis genes (*TaPAL33* and *OsCCR17*). This transcriptional response is coordinated with post-translational regulation involving suppression of PME activity and activation of pectin methylesterase inhibitors (PMEI). Collectively, these molecular adjustments lead to increased deposition of cellulose, hemicellulose, and lignin, elevated pectin methylesterification, subsequent cell wall thickening, and enhanced mechanical strength and thermotolerance. CWI: cell wall integrity; H_2_O_2_: hydrogen peroxide; ROS: reactive oxygen species; NO: nitric oxide; CNGCs: cyclic nucleotide-gated channels; CaM: calmodulin; LLGs: glycosylphosphatidylinositol-anchored proteins; FER: FERONIA (a member of the CrRLK1L receptor kinase family); THE1: THESEUS1 (a member of the CrRLK1L receptor kinase family); CSC: cellulose synthase complex; WAKs: wall-associated kinases; LRX: leucine-rich repeat extensins; RALF: rapid alkalinization factor; HSF: heat shock transcription factor; PMEs: pectin methylesterases; PMEI: pectin methylesterase inhibitor; *XTHs*: xyloglucan endotransglucosylase/hydrolase; TaPAL33: *Triticum aestivum* phenylalanine ammonia-lyase 33; OsCCR17: *Oryza sativa* cinnamoyl-CoA reductase 17. This figure was created by a online website BioRender. https://BioRender.com/633o967 (accessed on 22 May 2025).

The cell wall undergoes dynamic remodeling in response to biotic and abiotic stresses to modify and maintain its mechanical strength. Pathogen invasion induces the deposition of callose or lignin, forming a physical barrier that increases the rigidity of the cell wall and makes it less permeable [25]. Salt stress modifies hemicellulose, increases the degree of pectin methylation, and enhances pectin cross-linking, thereby maintaining turgor pressure in the cells [26]. At the early stage of nitrogen deficiency stress in cucumber (*Cucumis sativus*), several enzymes are activated, including pectin lyases (Csa1G049960), xyloglucan endotransglucosylase/hydrolases (Csa1G188680), pectinesterases (Csa7G447990; Csa7G343850), and expansin (Csa5G517210). This upregulation results in cell wall loosening and promotes cell expansion, facilitating the plant’s adaptation to nitrogen shortage [27]. In addition, the nitrate transporter *AtNRT1.8* in *Arabidopsis thaliana* shows significant co-expression with cell wall biosynthesis enzymes, such as pectin and xyloglucan synthases, suggesting that nitrate metabolism dynamically influences cell wall assembly by modulating the synthetic activity of these enzymes [28]. Heat stress induces partial thinning and loosening of the cell wall, a rapid response that is similar to the adjustments in wall architecture observed in desiccation-tolerant plants. This adjustment occurs through reduced hemicellulose branching and/or modified pectin cross-linking [29]. Thus, altered cell wall mechanical strength and plasticity caused by the change in the cell wall component and structure serve as a critical foundation for plant adaption to HS (Figure 1).

## 3. Cell Wall Expansion Mediates Heat Stress Sensing in Plants

Cell wall expansion is an important primary perceptual response to HS (Figure 1). Upon HS perception, plants rapidly reduce pectin methylesterification to enhance wall elasticity and porosity, facilitating cell expansion and stress adaptation [7]. Low-methylesterified pectin exhibits increased solubility and negative charge density, weakening cell–cell adhesion and loosening the wall architecture. Pectin is partially demethylesterified by PMEs. High temperatures of 35–65 °C cause an activation of PME activity and a decrease in pectin methylesterification in green bean and tomato [30]. Heat-activated PME activity is also involved in the removal of apoplastic Ca^2+^ that participates in HS signaling to induce HSP expression and cell wall remodeling for conferring thermoprotection [31]. Demethylesterified pectin also becomes more susceptible to polygalacturonase (PG)-mediated hydrolysis, dynamically adjusting the wall rigidity during pathogen responses [32]. Furthermore, the pectin content, PME activity, and pectin demethylesterification are involved in H_2_O_2_-induced cell expansion [33].

Heat stress alters the cellulose synthesis and architecture to promote cellular expansion. Heat stress inhibits cellulose synthase (CESA) activity, reducing cellulose deposition [34]. Concurrently, it modifies cellulose crystallinity—increasing lattice spacing and disorganizing microfibril alignment—thereby weakening the wall mechanics [35]. Heat stress disrupts cellulose biosynthesis by downregulating CESA genes (*CESA7* and *CESA6B*). For instance, overexpression of *MaNAC1* in banana upregulates *MaCESA6B/7*, thickening secondary walls under cold stress [36], whereas HS likely suppresses such genes, reducing cellulose crystallinity, which refers to the structural arrangement of cellulose molecules within a cellulose fiber. Additionally, HS remodels microtubule stability to reorient cellulose microfibrils, adjusting wall extensibility. Elevated expression of microtubule-associated proteins (RIC1) under HS may bundle microtubules, further lowering cellulose crystallinity and enhancing wall plasticity [29].

Heat stress disrupts the expression of hemicellulose- and lignin-related genes and damages cross-linking structures [37]. HS suppresses peroxidase activity, reducing lignin deposition and increasing wall flexibility. Concurrently, xyloglucan endotransglucosylase/hydrolases (XTHs) remodel the hemicellulose–cellulose network by cleaving and re-linking xyloglucan chains, facilitating turgor-driven expansion [38]. For example, cucumber hypocotyl elongation under HS is regulated by *CsSh5.1*, a xyloglucan galactosyltransferase [39]. Under HS, three *XTH* (*XTH11*, *XTH29*, and *XTH33*) genes are significantly upregulated in *Arabidopsis* roots, elongating xyloglucan (XG) chains to reinforce cell walls [40]. In maize, *ZmHSL* encodes a GH10-family endo-β-1,4-xylanase that specifically cleaves β-1,4-glycosidic bonds in xylan backbones. Reduced *ZmHSL* activity could decrease both the lignin and xylose content, leading to thinner xylem cell walls and impaired water transport. However, the mechanistic link between *ZmHSL* and cell wall remodeling remains unclear [41]. The *Zmhsl-1* mutant exhibits stunted growth and leaf curling under HS. Although RNA-seq analysis helps to reveal that the differentially expressed genes identified between Z58 and *Zmhsl-1* plants are mainly related to heat stress-responsive genes and unfolded protein response genes, the protein and regulation mechanism of *ZmHSL* involved in HS response requires further research [42].

Expansin is a protein involved in the elongation of the PCW and HS adaption. It functions by binding to the interface between cellulose microfibrils and xyloglucans in the cell wall, disrupting hydrogen bonding networks at these junctions. This process enhances the mechanical extensibility of the wall, thereby promoting cell expansion [43]. For instance, the expression of the potato expansin gene *StEXLB6* was significantly increased under HS, facilitating the expansion of the PCW [44]. Furthermore, transgenic wheat plants overexpressing the expansin gene *TaEXPA7-B* exhibit enhanced relaxation of the primary root cell wall, which promotes water absorption and confers greater salt tolerance [45]. Although research on expansin’s response to HS is limited, its role in maintaining the expansion of PCW under HS cannot be overlooked and warrants further investigation.

## 4. Cell Wall Thickening as an Adaptive Response to Heat Stress

Cell wall thickening is a crucial thermo-adaptive response in plants that enhances the mechanical strength and structural stability of the cell wall [34] (Figure 1). Heat stress-triggered cell wall thickening involves upregulation of biosynthesis-related genes to promote the accumulation of cell wall polysaccharides and lignin, coupled with downregulation of genes involved in cell wall degradation, collectively maintaining wall integrity [46,47]. Enhanced cross-linking among cell wall polysaccharides such as cellulose, hemicellulose, and pectin contributes to improved mechanical strength, while increased lignin deposition helps maintain cellular water homeostasis and alleviate high-temperature-induced water deficit. The table below summarizes reported cell wall-related genes and proteins, along with their functions under HS (Table 1).

The increase in methylesterified pectin contributes to enhanced cell wall thickness and mechanical strength. Pectin is synthesized in highly methylesterified form in the Golgi apparatus before undergoing partial demethylesterification by PMEs in the cell wall [56]. The mutation of PMEs results in a lower number of free carboxyl groups in the pectin molecule, which significantly impacts its interaction with Ca^2+^ [57]. For instance, the *pme53* mutant of *Arabidopsis* shows reduced calmodulin expression under HS, suggesting its role in wall remodeling via calcium signaling pathways to enhance thermotolerance [53]. The *Arabidopsis pme12* mutant had reduced total PME activity under HS, but its heat tolerance was instead enhanced, suggesting that changes in the activity of specific PME isozymes play a key role in HT acclimatization through local modifications to defend the cell wall or coordinate calcium signaling [47]. PME53 is classified as a type-II pectin methylesterase, while PME12 functions as a type-I pectin methylesterase. The structural differences between PME53 and PME12 might impact function divergence in HS. This finding suggested that selecting plant materials with high-methylesterified pectin content or reducing PME activity could improve heat tolerance through wall thickening. In contrast, continuous demethylation of pectin leads to the generation of a large number of negatively charged carboxyl groups that bind to Ca^2+^ and form an ‘egg box’ structure, further increasing the strength and thickness of the cell wall, which might indicate the dual roles of pectin methylesterification in both cell wall loosening and thickening [58]. HS also disrupts the dynamic balance between PMEs and their inhibitors (PMEIs), which determines the pectin methylesterification state [52]. Overexpression of *AtPMEI-1* or *AtPMEI-2* in *Arabidopsis* significantly reduced PME activity and increased the levels of pectin methylesterification [59]. In addition, under HS, pectin binds rapid alkalinization factor 1 (RALF1) and forms pectin-RALF-FER-LLG1 condensates through phase separation, activating the receptor kinase FERONIA (FER). FER senses cell wall integrity (CWI) by preferentially binding de-esterified pectin, subsequently initiating ROP6 signaling to regulate morphogenesis and promote the recovery from heat-induced growth inhibition in *Arabidopsis* [54,60,61]. Therefore, the methylesterification of pectin, regulated by the balance between PME and PMEI, along with the phase separation between pectin and feruloyl ester (FER) receptors, all influence cell wall thickening and hypersensitive responses. It is a quite complex network between pectin and other proteins that needs further investigation.

Heat stress activates the expression of cellulose synthase (*CESAs*) genes, promoting cellulose biosynthesis and deposition, directly contributing to cell wall thickening. Plants overexpressing *ZmCESA2* exhibited enhanced survival rates under HS, and cell wall composition analysis revealed increased cellulose content, which plays a crucial role in providing cell wall strength [11]. Previous studies have demonstrated that plants overexpressing *ZmCESA2* exhibit enhanced cold tolerance [62], suggesting that elevated cellulose content provides universal benefits for plant survival under varying temperature conditions. Temperature stress may indirectly regulate CESA gene expression through the activation of specific transcription factors, thereby promoting cellulose microfibril synthesis and deposition. Research indicates that overexpression of both PCW-associated (*OsCESA5*) and SCW-associated (*AtCESA7*) cellulose synthase genes can increase cell wall thickness through distinct mechanisms [63,64]. However, the functional differentiation between primary and secondary cell walls in plant responses to HS requires further investigation.

Lignin, a complex phenolic polymer, also significantly enhances the mechanical strength of cell walls and improves stress resistance in plants [49,65]. In poplar, HS activates key enzymes involved in lignin biosynthesis, including phenylalanine ammonia-lyase (PAL), cinnamoyl-CoA reductase (CCR), and laccase (LAC), thereby promoting lignin synthesis and deposition [65]. Transcriptome analysis of wheat under different durations of heat treatment identified *TaPAL33* as a heat-responsive gene. Overexpression of *TaPAL33* in wheat increased PAL activity, alleviated heat-induced oxidative stress, and enhanced thermotolerance, though it also elevated lignin content while suppressing growth [49]. Research on heat tolerance mechanisms during rice reproductive development revealed that *OsCCR17* expression is upregulated by both high temperature and hormonal signals [50]. Genetic manipulation of the vesicle-associated membrane protein VAMP726 from maize and *Arabidopsis* not only affected lignin monomer composition in sporopollenin but also pollen resistance to heat and UV radiation [51]. Under combined heat and drought stress, secondary wall thickening mediated by lignin and suberin deposition reduces water loss and improves structural stability [66]. Exogenous salicylic acid (SA) enhances this protective mechanism by upregulating the enzymatic activity of cinnamoyl-CoA reductase (CCR) and phenylalanine ammonia-lyase (PAL), leading to significantly increased lignin accumulation in maize under HS [67]. NAC transcription factors broadly participate in stress responses; research has demonstrated that the membrane-associated *LlNAC014* in lily activates the expression of *LlDREB2B* (*Dehydration-Responsive Element-Binding 2B*), an upstream regulator of HSFs, which in turn upregulates *LlHSFA3B* to positively modulate thermotolerance [68]; *EgNAC141* enhances lignin deposition in *Eucalyptus*; *NST1* (secondary wall thickening promoting factor 1) activates the expression of lignin biosynthetic genes in *Arabidopsis*; and *PtrWND2B* drives ectopic lignin accumulation by activating lignin biosynthetic genes in poplar [69,70]. However, whether NAC transcription factors participate in lignin biosynthesis under heat stress remains largely unexplored.

## 5. The Regulation of Heat Stress Response by Maintaining Cell Wall Integrity

### 5.1. The Role of Cell Wall Integrity in Signaling Heat Stress

Plant cell wall integrity (CWI) refers to the stability and functionality of the cell wall structure, playing a critical role in plant growth, development, and environmental interactions [11,71]. It involves the de novo synthesis of cell wall components, physical remodeling of the wall, and maintenance of physiological functions, as well as proper signal transduction and response mechanisms [72]. CWI further depends on the balanced composition of polysaccharides (cellulose, hemicellulose, and pectin) and other components (proteins and lignin). Importantly, it is essential for maintaining cellular water retention, particularly under HT and drought stress conditions. The wall structure must retain appropriate porosity to allow the efficient transport of water, nutrients, and signaling molecules between cells. Under HS, changes in the composition and structure of the plant cell wall activate the CWI sensing system, triggering downstream regulatory factors that modulate wall remodeling to mitigate heat-induced damage and enhance thermotolerance [73].

During HS, alterations in the structural composition of plant cell walls can result in mechanical damage, which subsequently triggers the accumulation of key signaling molecules, including ROS and nitric oxide (NO) (Figure 1). These signaling molecules are essential for the induction of heat-responsive genes, as well as genes involved in cell wall synthesis and modification. For example, critical enzymes associated with this phenomenon include respiratory burst oxidase homologs (RBOHs), which are responsible for ROS production, and various peroxidases that modulate cell wall components [74]. GT-1 is a trihelix transcription factor that can bind to a cis-acting element known as BoxII (GGTTAA) in *Arabidopsis*. Under HS, S-nitrosylation of GT-1 enhances its binding to nitric oxide (NO)-responsive elements in the *HSFA2* promoter. This finding reveals that GT-1 is a long-sought mediator linking NO signal perception to the activation of cellular heat responses [75]. Through these coordinated pathways, CWI is maintained, ultimately enabling plant adaptation to elevated temperatures [5,76].

### 5.2. Perception of the Change in Cell Wall Integrity

The content and modification of pectin serve as critical signals in the perception of CWI. Homogalacturonan (HG), a major structural component of the cell wall, also functions as a signaling molecule that monitors CWI and is recognized by specific plasma membrane-localized receptors to initiate intracellular signaling [58]. Mutations in the pectin methyltransferase QUASIMODO2 (QUA2) in *Arabidopsis* impair pectin biosynthesis and disrupt the mobility of cellulose synthase complexes (CSCs) at the plasma membrane, demonstrating that pectin strands are essential for organizing cellulose microfibrils by spatially guiding CSC trajectories. These defects compromise CWI, resulting in inhibited hypocotyl elongation and epidermal cell adhesion abnormalities [77]. Using ^13^C labeling and solid-state NMR spectroscopy, Kirui provided molecular-level evidence for pectin–cellulose interactions, offering novel insights into how cell wall architecture and integrity govern plant growth and development [78].

The leucine-rich repeat extensin (LRX) glycoproteins anchor to the cell wall via their C-terminal arabinosylated extensin domain, while their N-terminal LRR domain interacts with RALF peptides, ligands of FERONIA (FER), thereby participating in the perception of CWI [55]. The plasma membrane receptor kinases WAK (wall-associated kinase) and WAKL (wall-associated kinase-like) recognize oligogalacturonides released from pectin degradation, activating the MAPK (mitogen-activated protein kinase) cascade to initiate HS sensing and response [79,80]. Additionally, studies have demonstrated that the assembly, trafficking, and localization of the cellulose synthase complex (CSC) contribute to plant adaptation to salt stress [10,81].

### 5.3. Molecular Mechanisms Underlying the Maintenance of Cell Wall Integrity Under Heat Stress

#### 5.3.1. Transcriptional Regulation

Heat shock factors (HSFs) play pivotal roles in modulating cell wall-related gene expression under HS. In rose plants, six class A HSF genes exhibit significant upregulation under heat stress and respond to ABA, JA, and H_2_O_2_ signaling pathways to mitigate thermal damage [82]. HSFs are essential for cell survival by coordinating crosstalk between CWI pathways and heat shock responses through the activation of HSPs [83]. Furthermore, upregulated heat-responsive genes (*HSP12*, *HSP26*) demonstrate functional linkage with cell wall polysaccharide biosynthesis (chitin and β-1,3-glucan) [84]. Thermotolerance assays using *TaPIF4* mutant and phosphomimetic overexpression lines in wheat revealed that phosphorylated *TaPIF4* substantially enhances heat resistance [82]. In maize, *ZmHSF20* functions upstream of *ZmHSF4*, which directly activates *ZmCesA2* expression to enhance cellulose biosynthesis, thereby reinforcing cell wall mechanical strength and thermotolerance [11]. Necrotic upper tips1 (nut1) encodes a NAC transcription factor in maize, which localizes to the developing protoxylem to activate the genes necessary for reinforcing the secondary cell wall. NUT1 mediates synergistic responses to combined heat and drought stresses by directly regulating cellulose biosynthesis and xylem cell wall thickness during protoxylem development in flowering [48]. *ZmNAC074* of maize is induced by multiple stresses, particularly in heat-treated leaf tissues during the seedling stage, and potentially enhances thermotolerance through the dual activation of ROS-scavenging genes and heat shock response (HSR)/unfolded protein response (UPR) pathways [85,86].

#### 5.3.2. Cross-Regulation of Hormone Signaling

Plant hormones, particularly abscisic acid (ABA), auxin, and cytokinin, play pivotal roles in cell wall adaptation mechanisms. ABA-induced defense genes may enhance mechanical strength through increased wall cross-linking or lignin deposition. Cell wall damage or remodeling activates the CWI system, modulating gene expression (defense- or growth-related genes) via hormone signaling pathways such as jasmonate (JA) and brassinosteroids (BRs). Under HS, BRs likely coordinate cell elongation and wall remodeling [87]. In *Arabidopsis*, THESEUS1 (THE1) is a key regulator of wall mechanical strength and turgor, revealing its synergistic interplay with JA/ABA to control turgor pressure and wall reinforcement [88].

## 6. Conclusions and Perspective

Heat stress represents a critical environmental factor that adversely affects plant growth, development, and ultimately yield and quality. As a distinctive structural feature distinguishing plants from animals, the cell wall serves not only as the primary barrier for environmental sensing but also plays an indispensable role in signal transduction under HS. This review systematically elaborates on the dynamic changes in the composition, structure, and mechanical properties of plant cell walls under HS, the molecular regulation of cell wall thickening in response to HS, as well as the pivotal role of CWI in both HS perception and signal transduction (Figure 1). Although recent studies have identified and characterized the functional importance of specific cell wall components and genes in HS responses, a series of fundamental scientific questions remain to be further elucidated.

### 6.1. Which Components of the Plant Cell Wall Are Crucial for Responding to Heat Stress, and How Do the Mechanisms Differ Among Various Plant Species?

The plant cell wall is composed of multiple components including cellulose, hemicellulose, lignin, and pectin, yet their respective roles and coordinated regulatory mechanisms in response to HS remain to be systematically elucidated. The development of plant cell walls exhibits spatiotemporal specificity, encompassing both primary and secondary cell walls, whose dynamic remodeling under HS requires in-depth investigation [24]. Whether there is a sequence of changes in different cell wall components in response to HS is still unknown. Furthermore, the molecular mechanisms underlying how these structural characteristics balance cellular expansion and thickening under heat stress warrant further exploration. In particular, the dual roles of pectin methylesterification in both cell wall loosening and thickening during thermotolerance responses should be key research priorities. During the initial stage of heat stress, the loosening of the cell wall facilitates the influx of Ca^2+^ from the apoplast into the cytoplasm. This increase in the cytoplasmic Ca^2+^ concentration acts as a signal, initiating the plant’s response to heat stress. However, under prolonged heat stress, plants require the tightening of the cell wall to better withstand these conditions. One mechanism for this involves the binding of Ca^2+^ to demethylated pectin, forming an ‘egg box’ structure that enhances the strength and thickness of the cell wall [58]. The spatiotemporal expression of genes encoding pectin methylesterases (PMEs) and pectin methylesterase inhibitors (PMEIs) may function as the dichotomy to regulate calcium homeostasis and dictate the specific roles of Ca^2+^ in heat stress sensing and response.

The mechanisms for cell wall reconstruction in response to HS also exhibit variability among different types of plants, such as monocots and dicots, as well as herbaceous and woody plants. These plants typically follow similar pathways in their HS sensing and response as described in Figure 1. However, due to the differences in cell wall structure and composition, as well as the diversity of the transcriptional regulatory networks governing cell walls, the expression of relevant genes is spatially and temporally specific [17,18,19,20]. These specificities influence the extension and thickness of the cell wall, leading to differences in the adaptability of various species to HS environments. For example, some tall plants are better adapted to tropical regions due to their more effective cell wall reconstruction mechanisms for coping with heat stress.

### 6.2. What Are the Sensing Mechanisms of Plant Cell Wall Integrity Under Heat Stress?

Under HS, plants undergo significant alterations in cell wall composition and architecture, which subsequently activate the CWI surveillance system. This activation initiates a downstream signaling cascade involving key regulatory switches that orchestrate cell wall remodeling processes, ultimately enabling plants to mitigate heat-induced damage and acclimate to elevated temperatures. Although several CWI-sensing proteins have been identified, the molecular mechanisms underlying HS perception and signal transduction remain poorly understood [73]. While HS triggers the specific expression of certain HSPs and HSFs, the precise subset of genes that modulate cell wall biosynthesis in response to HS requires systematic identification.

### 6.3. How Does the Plant Cell Wall Balance Growth and Stress Resistance Under Heat Stress?

Plants cope with HS through precisely regulated cell expansion and wall thickening. Elevated temperatures may enhance cell wall plasticity to facilitate thermal adaptation, while concurrent wall thickening reinforces mechanical strength and structural integrity, thereby protecting the cellular architecture and function from heat-induced damage. However, the molecular mechanisms underlying the cell wall’s coordination of growth and thermotolerance remain incompletely characterized [89]. Furthermore, as HS imposes substantial energetic demands, future research should address how plants optimize resource allocation to sustain stress resilience, representing a critical frontier in understanding plant adaptation strategies.

### 6.4. Genetic Engineering of Cell Walls to Augment Plant Heat Stress Adaptation

The plant cell wall comprises a sophisticated hierarchical regulatory network that can be genetically modified through three primary approaches. Engineering key genes involved in cell wall component biosynthesis, such as *Cellulose Synthase* and *Phenylalanine Ammonia-lyase*, directly modulates cell wall thickness and ultrastructure. Optimizing cell wall-modifying enzymes, including xyloglucan endotransglucosylases/hydrolases (XTHs), pectin methylesterases (PMEs), and glycoside hydrolases (GHs), can significantly alter the mechanical properties of the cell wall. Additionally, these modifications may lead to the production of stress-responsive oligosaccharide signaling molecules such as cellodextrins, xyloglucan, homogalacturonan, and oligo-galacturonic acids [56,90]. *MYBs* and *NACs* transcription factors that coordinate multiple cell wall synthesis pathways enable comprehensive regulation of wall integrity [91]. Despite these advances, the molecular genetic mechanisms underlying cell wall-mediated thermotolerance require systematic investigation. Comprehensive profiling of these regulatory components during distinct heat stress phases—from initial perception through memory formation—will identify critical stress-responsive determinants. Functional validation through precision genome editing and transgenic approaches will elucidate how cell wall integrity contributes to thermal resilience, providing both fundamental insights and genetic resources for crop improvement. Concurrently, developing high-throughput phenotyping platforms for heat stress responses coupled with advanced cell wall analytics will enable genome-wide association studies to identify quantitative trait loci (QTLs) linking thermotolerance with specific wall modifications. This integrated strategy will facilitate the discovery of superior haplotypes from diverse germplasm collections, ultimately accelerating the development of climate-resilient crop varieties with optimized yield potential under HS.

## Figures and Tables

**Table 1:** Functions of reported cell wall genes and proteins in heat stress response.

Cell Wall Components	Associated Genes and Enzyme Proteins	Name of the Species	Regulatory Role Under Heat Stress	References
Cellulose	*ZmCESA2*	*Zea mays*	Promotes cell wall thickening, positive regulation of HS	[11]
	*ZmHSF4*	*Zea mays*	Direct activation of ZmCesA2 expression, positive regulation of HS	[11]
	*nut1*	*Zea mays*	Directly affects cellulose biosynthesis, positive regulation of HS	[48]
	RIC1	*Arabidopsis thaliana*	Enhances cell wall extensibility, positive regulation of HS	[29]
Hemicellulose	*XTHs*	*Arabidopsis thaliana*	Enhances cell wall reinforcement, positive regulation of HS	[40]
	*CsSh5.1*	*Cucumis sativus*	Encodes galactosyltransferase for xyloglucan, positive regulation of HS	[39]
	*ZmHSL*	*Zea mays*	Promotes cell wall thickening, positive regulation of HS	[42]
Lignin	*TaPAL33*	*Triticum aestivum*	Promotes lignin deposition, positive regulation of HS	[49]
	*OsCCR17*	*Oryza sativa*	Promotes lignin deposition, positive regulation of HS	[50]
	VAMP726	*Zea mays*	Promotes the transport of lignin monomers, positive regulation of HS	[51]
Pectin	*PMEIs*	*Arabidopsis thaliana*	Blocks PME activity, maintaining wall flexibility, positive regulation of HS	[52]
	*AtPME12/AtPME34/AtPME53*	*Arabidopsis thaliana*	Promotes pectin demethylesterification, negative regulation of HS	[46,47,53]
	RALFs	*Arabidopsis thaliana*	Pectin binding triggers phase separation of RALF-FER-LLG1 signaling modules, positive regulation of HS	[54]
	LRX	*Arabidopsis thaliana*	Perception of plant cell wall integrity through LRR domain–RALF peptide interactions, positive regulation of HS	[55]

*ZmHSF4*: maize heat shock transcription factor 4; *nut1:* necrotic upper tips1; *XTHs*: xyloglucan endotransglucosylase/hydrolase; *ZmHSL*: maize *Zea mays* heat-sensitive leaves; *TaPAL33*: *Triticum aestivum* phenylalanine ammonia-lyase 33; *OsCCR17*: *Oryza sativa* cinnamoyl-CoA reductase 17; VAMP726: vesicle-associated membrane protein 726; PMEs: pectin methylesterases; PMEI: pectin methylesterase inhibitor; RALF: rapid alkalinization factor; LRX: leucine-rich repeat extensins; HS: heat stress.

## Data Availability

Not applicable.
